# MRL Strains Have a BAFFR Mutation without Functional Consequence

**DOI:** 10.1371/journal.pone.0154518

**Published:** 2016-05-05

**Authors:** Windy R. Allman, Lunhua Liu, Adam S. Coleman, Mustafa Akkoyunlu

**Affiliations:** Laboratory of Bacterial Polysaccharides, Division of Bacterial Parasitic and Allergenic Products, Center for Biologics Evaluation and Research, U.S. Food and Drug Administration, Silver Spring, Maryland, 20993–0002, United States of America; INSERM-Université Paris-Sud, FRANCE

## Abstract

It has been shown that B cell activating factor receptor (BAFFR) is critical for B cell development and survival. In this study, we sought to evaluate the expression and function of BAFFR across multiple stains of mice that vary in their potential to develop systemic autoimmune disease. The inability of a commercial antibody to bind to BAFFR in the autoimmune prone mouse strains, MRL and MRL/Lpr led to the discovery of a mutation in *TNFRSF13C* gene (encoding BAFFR) that resulted in a Pro44Ser substitution in the N-terminus near the BAFF binding site in these strains. To define the biological consequences of mutant BAFFR, we compared the expression and activity of BAFFR in MRL and MRL/Lpr mice to BALB/c, which express the consensus version of *TNFRSF13C*. B cells from MRL and MRL/Lpr mice expressed mutant BAFFR on surface and were capable of responding to BAFF as exhibited by BAFF-mediated reduction in apoptosis and NF-κB2 activation. Signaling through MAPK ERK1/2 was not significantly induced by BAFF in MRL/Lpr mice; however, MAPK ERK1/2 signaling was intact in MRL mice. The inability of MRL/Lpr B cells to significantly activate ERK1/2 in response to BAFF was due to the high basal activity of the signaling pathway in these cells. In fact, basal activity of ERK1/2 in B cells correlated with the degree of autoimmune susceptibility exhibited by each strain. In addition, aged MRL/Lpr mice with severe autoimmune disease had high BAFF levels, low surface BAFFR, and high basal NF-κB2 activation, a pattern which is attributed to the high frequency of antibody secreting cells. We conclude that P44S BAFFR mutation does not hinder BAFFR function or enhance B cell activity in MRL/Lpr and MRL mice and that other susceptibility loci on the MRL background contributed to the hyperactivity of these cells.

## Introduction

Systemic lupus erythematosus (SLE) is a potentially life threatening autoimmune disorder characterized by chronic inflammation mediated by immune complexes capable of damaging multiple organs [1, 2]. The etiology of the disease is not clear. However, both environmental and genetic susceptibility are thought to play a role in the failure to maintain tolerance [3, 4]. SLE is associated with defects in B and T cell tolerance characterized by a diverse repertoire of antibodies against nuclear antigens such as dsDNA, ssRNA, RNA, nucleosomes and chromatin [1, 2]. MRL/Lpr mice are widely used as an animal model for SLE due to several common features of disease such as: the production of anti-nuclear antibodies, the development of immune complex mediated glomerulonephritis, splenomegaly, lymphadenopathy, and a greater disease occurrence among females [2, 5, 6]. The acceleration of SLE in MRL/Lpr mice is due to a spontaneous mutation that leads to a functionally inactive form of Fas, a death receptor capable of inducing apoptosis in activated lymphocytes [7, 8]. MRL mice, which express functional Fas, develop a mild and delayed lupus disease [9]. Thus, it appears like factors which control lymphocyte survival promote autoimmunity [10].

The enhanced expression of B cell activating factor of the TNF family (BAFF) has been implicated in the survival of autoreactive B cells in SLE [11, 12]. Transgenic mice over-expressing BAFF have increased numbers of circulating B cells and develop lupus like disease [10]. Another closely related cytokine, a proliferation-inducing ligand (APRIL), is also highly expressed in patients with SLE [13, 14]. Both BAFF and APRIL are members of the TNF family of ligands, which act as key mediators of B cell survival and plasma cell generation [15]. Both of these cytokines bind the receptors transmembrane activator and calcium-modulator and cytophilin ligand interactor (TACI) and B cell maturation antigen (BCMA), while BAFF receptor (BAFFR) binds exclusively to BAFF [16]. The Food and Drug Administration has approved Belimumab, an anti-BAFF antibody, as a treatment for SLE due to its efficacy in reducing disease activity and re-occurrences, called flares, in patients with active SLE [17]. As in human SLE, anti-BAFF antibodies were effective in reducing disease severity in mouse models by decreasing numbers of B cells and antibody secreting cells [18, 19]. However, it is unclear which receptor mediates autoimmune development through BAFF/APRIL signaling.

Since BAFFR is critical for the selection and survival of mature B cells, the elevated serum BAFF observed in SLE is likely promoting the survival of mature autoreactive B cells by inducing chronic BAFFR signaling [20–22]. BAFFR is predominately expressed by mature naïve and memory B cells [16, 23]. BAFFR stimulation results in the activation of alternative NF-κB2 pathway, which induces the expression of anti-apoptotic molecules, such as Bcl-2 and Bcl-X_L_ [20, 24–26]. BAFFR stimulation also suppresses the accumulation of pro-apoptotic Bcl-2 family member BIM via activation of the MAPK (ERK1/2) pathway [27, 28]. The BAFFR knock-out mouse has reduced late transitional and mature B cells, which corresponds to impaired serum antibody titers [29]. However, the A/WySnJ strain mouse, which has a spontaneous mutation in the BAFFR gene, *TNFRSF13C*, develops a lupus-like syndrome [11, 22]. In the A/WySnJ strain, the mutation in BAFFR, also called the B-cell maturation defect-1 (Bcmd-1), does not prevent BAFF binding but leads to diminished signaling due to an insert in the signaling domain at the C-terminus [22, 24, 30]. A/WySnJ mice develop a late onset of disease associated with high titers of circulating IgM and IgG to dsDNA and renal pathology due to immune complex deposition in the glomeruli [30]. BAFFR Bcmd-1 was necessary for the development of disease since BAFFR knock-out A/WySnJ mice do not develop SLE. However, C57BL/6 mice do not develop lupus disease if they express mutant BAFFR Bcmd-1 which suggests the contribution of multiple genetic factors in the development of SLE in A/WySnJ mice [31]. Yet other strains of mice such as NZM 2328 spontaneously develop SLE, with similar levels of anti-dsDNA IgG even in the absence of BAFFR [32].

In this study, we sought to assess the status of BAFFR expression and function in MRL/Lpr mice. We discovered a mutation in *TNFRSF13C* that resulted in a proline to serine substitution in the extracellular domain of BAFFR adjacent to the binding site of BAFF, a mutation that is carried by both MRL/Lpr and MRL strains. Further studies showed that the proline to serine substitution did not hamper BAFF activity mediated by BAFFR in the MRL background. Disease in MRL/Lpr was accompanied by high levels of BAFF in vivo, low BAFFR surface expression on B cells, increased peripheral antibody secreting cells, and elevated activation of alternative NF-κB2; which indicated in vivo BAFF activation of BAFFR. We conclude that this BAFFR mutation does not hamper BAFF function or lead to heightened B cell activity in MRL/Lpr and MRL mice and that other susceptibility loci on the MRL background contribute to the hyperactivity of these cells.

## Materials and Methods

### Mice

MRL/MpJ-Fas^*lpr*^/J (will be referred to as MRL/Lpr throughout the manuscript) and MRL/MpJ (will be referred to as MRL throughout the manuscript) mice, were purchased from The Jackson Laboratory (Bar Harbor, ME). Adult BALB/c mice were purchased from Charles River Laboratories (Wilmington, MA). Mouse experiments were approved by the US Food and Drug Administration/Center for Biologics Evaluation and Research Institutional Animal Care and Use Committee (permit number 2002–31). As approved in the protocol, mice showing advanced SLE disease symptoms such as severe skin lesions characterized by >5% of the skin surface, stress symptoms manifested with increased respiratory rate with an abdominal component in breathing, loss of response to external stimuli, and severe lymphadenopathy characterized by easily visible lymph nodes interfering with mobility were euthanized early as a humane endpoint.

### Reagents and Antibodies

Antibodies against mouse cell markers and the isotype controls used in flow cytometry assay were as follows: BAFFR-ATTO 488 (9B9) and rat IgG2b-ATTO 488 (A-1) from Enzo Life Sciences, Inc (Farmingdale, NY); BAFFR-FITC (clone 7H22-E16), rat IgG1-FITC and fixable viability dye eFlour 780 from (eBioscience, San Diego, CA); CD138-PE (281–2), rat IgG2a-PE (R35-95), and BV605-IgD (11-26C.2a) and BV605-rat IgG2a,λ (B39-4) from BD Biosciences/BD Pharmingen (San Jose, CA); PerCP/Cy5.5-IgM (RMM-1), PerCP Cy5.5 Rat IgG2a,κ (RTK2758), Pacific Blue-CD19 (6D5), Pacific Blue Rat IgG2a,κ (RTK2758), CD93-APC (AA4.1), rat IgG2b-APC (RTK4530) from BioLegend (San Diego, CA); and Annexin V-Alexa Fluor 647 (Invitrogen, Grand Island, NY). Propidium iodide (0.5 μg/ml) was used to identify dead cells (BD Biosciences/BD Pharmingen). B cell isolation Kits were purchased from Miltenyi Biotec Inc (San Diego, CA) to negatively select B cells from spleen as described previously [33, 34]. BAFF was purchased from R&D Systems. Antibodies used in Western blot analysis were as follows: NF-κB2 p100/p52, Phospho-p44/42 MAPK (ERK1/2) (Thr202/Tyr204) (D13.14.4E) XP-HRP, p44/42 MAPK (ERK1/2) (137F5), and β-actin-HRP (13E5) (Cell Signaling Technologies, Danvers, MA).

### Determination of B cell Subsets and BAFFR expression by flow cytometry

Single cell suspensions of spleen were obtained by mechanic dissociation of tissue through a 70-μM filter. Red blood cells were then lysed using ACK lysing buffer (Lonza, Wallersville, MD). Spleen cells (10^6^) were stained with fluorescent anti-mouse antibodies after blocking CD16/CD32 with Fc Block. B cells were gated using CD19 antibodies and subsequently analyzed for fluorescence intensity of BAFFR staining on B cell subsets using a LSRII instrument (BD Biosciences). Where indicated B cells (CD19^+^) were gated for splenic B cell subsets based on CD93, IgM, IgD and CD138 expression, using the previously described gating strategy published by Uslu and colleagues [34]. Data were analyzed using FLOWJO version 7.2.5 for PC (Tree Star, Ashland, OR) and Prism 5 (GraphPad Software, La Jolla, CA).

### Genetic Analysis of BAFFR (*TNFRSF13C*)

DNA was extracted from tail samples of at least 5 female mice per strain using the QIAamp DNA Mini kit (Qiagen, Hilden, Germany). Primers were designed flanking each of the exons in the mouse BAFFR gene *TNFRSF13C*: exon 1 forward, TGG GCG CCA GGA GAC T; exon 1 reverse, CCA GGG TCA GCA GAG GAG; exon 2 forward, CAT CCC TTT ATC CCC CTC AT; exon 2 reverse, CGG CAG TTC TCA CCT TGC TG; exon 3 forward, CTG TGA TTC CGT CCT TCC TG; exon 3 reverse, GAA GGG CCA AGT TCT TTC C. Each exon was amplified using conventional PCR, and the amplicons were purified using the MinElute PCR purification kit (Qiagen). The purified amplicons were sequenced bi-directionally using the same primers by the FDA Facility for Biotechnology Resources core facility, with the resulting sequences analyzed using Chromas Lite (http://www.technelysium.com).

### Quantitative Real-Time PCR

B cells were isolated via negative magnetic selection using B cell isolation kit (Miltenyi Biotec).

Total RNA was extracted from splenic B cells of female mice at two months of age using the RNeasy Mini kit (Qiagen). Five hundred nanograms of total RNA was reverse-transcribed into cDNA using random hexamers with the Taqman Reverse transcription kit (Invitrogen). Gene expression of BAFFR and GAPDH were determined using Taqman Gene Expression assays, Mm00840578_g1 and Mm99999915_g1, using a CFX96 Touch Real-Time System (BioRad, Hercules, CA). Relative expression values were determined by the 2^-ΔCt^ method where samples were normalized to GAPDH expression.

### Apoptosis Assay

Flow cytometry sorted B cells were isolated via negative magnetic selection using B cell isolation kit (Miltenyi Biotec). B cells were plated at 2x10^6^ cells/ml in 48-well flat bottom plates. Cells were stimulated in RPMI 1640 complete medium alone or with increasing concentrations of BAFF. After 48 hours of stimulation, the frequency of live cells was determined by flow cytometry. Briefly, cells were washed twice with ice cold PBS and resuspended in Annexin V binding Buffer (10 mM HEPES, 140 mM NaCl, and 2.5 mM CaCl_2_, pH 7.6) at 1x10^6^ cells/ml. Cells were incubated for 15 minutes at room temperature with Annexin V-Alexa Fluor 647 and propidium iodide. Volume was brought to 400 μl with annexin V binding buffer and read using S1000EXi flow cytometer (Stratedigm, San Jose, CA). Data were analyzed using FLOWJO version 7.2.5 for PC (Tree Star) and Prism 5 (GraphPad Software).

### Immunoblotting

Flow cytometry sorted B cells were plated at 2x10^6^ cells/ml in 96-well flat bottom plates. Cells were stimulated in RPMI 1640 complete medium alone or with increasing concentrations of BAFF. After 24 hours of stimulation, the processing of NF-κB2 p100 and phosphorylation of ERK1/2 was determined by Western blot analysis. Cells were first washed with PBS and then lysed in 1X NuPAGE LDS sample buffer supplemented with NuPAGE sample reducing agent (Invitrogen). Lysates were separated on 4–20% Mini-PROTEAN TGX gels (BioRad) and transferred to nitrocellulose using the iBlot transfer system (Invitrogen). The membranes were probed using anti- NF-κB2 p100/p52 or anti-Phospho ERK1/2 (T202/Y204). Goat anti-rabbit IgG-HRP were used to detect antibody binding (Cell Signaling Technology). Blots were then stripped and probed with anti-β actin-HRP or anti-ERK1/2-HRP antibodies (Cell Signaling Technology). Blots were developed using LumiGLO reagent (Cell Signaling Technology) and visualized using Innotech FluorChem Imaging System (Protein Simple, Santa Clare, CA).

### Determination of sera BAFF concentration

Sera from 2 and 5 month old female mice were diluted in assay diluent at two-fold serial dilutions starting at 1:5 to 1:500. Sera BAFF levels were measured using Quantikine^®^ Colorimetric Sandwich ELISAs (R&D Systems) according to the manufacturer’s protocol. BAFF concentrations in triplicate serum samples were determined by serial dilution of BAFF standard provided by kit.

### Clinical evaluation of mice using urinalysis

Urinalysis of female mice was performed at the ages of 2 and 5 month to determine proteinuria by dipstick detection with Multistix^®^ (Siemens Healthcare Diagnostics). Proteinuria measurements were categorized as follows; Grades 0 and 0.5+ were considered normal, Grades 1+ and 2+ were defined as proteinuria positive with mild to moderate impairment of kidney function, and Grades 3+ and 4+ represented severe proteinuria and impaired kidney function.

### Detection of total IgM, IgG and anti-dsDNA antibodies in sera

Sera from 2 and 5 month old female mice were diluted in buffer (PBS, 1% BSA, 0.05% Tween 20) at two-fold serial dilutions starting at 1:5 to 1:40,000. Anti-dsDNA IgG levels were determined using a modified version of previously described sandwich ELISA method [5]. Briefly, calf thymic DNA (Sigma) was coated on a 96-well microtiter plate (Dynatech Immulon 4 HBX; Dynatech Labs., Chantilly, VA) at 0.5 μg/ml with 0.1 M of carbonate bicarbonate buffer (pH 9.6) overnight at 4°C. Plates were blocked for 30 minutes at room temperature with 5% BSA in PBS and then washed with 0.05% Tween 20 PBS. Sera were added to plate in triplicate and incubated at 37°C for 2 hours. Plates were washed with 0.05% Tween 20 PBS and HRP conjugated anti-IgG antibody diluted at 1:1000 in 5% BSA was added to each well. Plates were incubated for 1 hour at room temperature. Finally, plates were washed with 0.05% Tween 20 PBS and measured at 405 nm absorbance after developing with ABTS Simple Solution (Invitrogen).

### Statistical analysis

Data from groups were compared using GraphPad Prism, Version 6 software (GraphPad Software, San Diego, CA) and analyzed by the Student’s t test (two tailed) and the Mann-Whitney U test where data distribution was non-normal.

## Results

### BAFFR is mutated in MRL and MRL/Lpr mice

**Fig 1:**
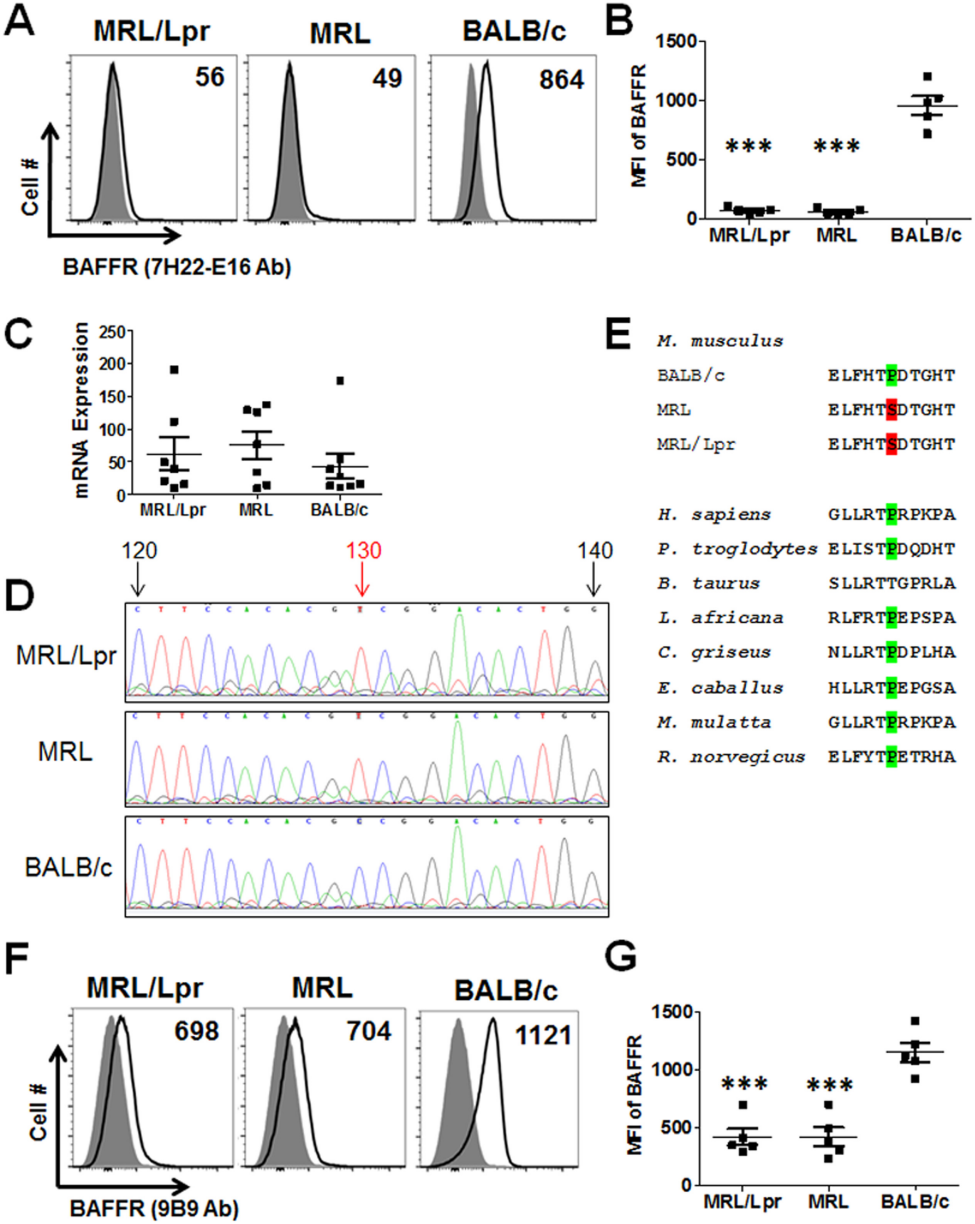
Identification of the BAFFR P44S mutation in MRL and MRL/Lpr strains in 2 month old mice. (A) Histograms of BAFFR expression on CD19^+^ splenic B cells determined by flow cytometry using the monoclonal antibody clone 7H22-E16. The mean fluorescence intensity (MFI) of B cells expressing BAFFR is indicated in each histogram. Filled area shows isotype control antibody and open line indicates the intensity of staining for BAFFR. Representative data from each strain are shown. (B) MFI ± SD of BAFFR expressing B cells determined by flow cytometry. Data shown are from 5 female mice per group. *** p ≤ 0.001 compared to BALB/c mouse. (C) Expression of BAFFR mRNA in purified B cells is shown. Relative gene expression of samples compared to GAPDH ± SD was plotted (n = 9 to10). (D) *TNFRSF13C* was sequenced and a cytosine to thymidine transition at position 130 was identified. (E) BAFFR amino acid sequence alignment of multiple mammalian species including the mouse strains BALB/c, MRL, and MRL/Lpr is shown. The alignment indicated that an evolutionary conserved proline (P) at codon 44 was substituted for a serine (S) in the extracellular domain. (F) Histograms of BAFFR expression on splenic B cells determined by flow cytometry using the monoclonal antibody clone 9B9. MFI of B cells expressing BAFFR is indicated. Filled area shows isotype control antibody and open line indicates the intensity of staining for BAFFR. Representative data from each strain are shown. (G) MFI ± SD of BAFFR on B cells determined by flow cytometry. Data shown are from 5 female mice per group. *** p ≤ 0.001 compared to BALB/c mouse.

Previously, we have shown that splenic B cells express high levels of surface BAFFR in BALB/c mice using the rat monoclonal anti-BAFFR antibody clone 7H22-E16 [35]. To begin assessing the status of BAFFR expression during SLE development, we compared BAFFR levels on B cells from 2-month old autoimmunity-prone MRL/Lpr and MRL strains to normal BALB/c mice using flow cytometry. Splenocytes were gated using the pan-B cell marker, CD19, and the frequency of BAFFR expressing B cells was analyzed. To our surprise, we failed to detect BAFFR on the surface of MRL/Lpr or MRL cells using anti-BAFFR (7H22-E16) antibody, which bound B cells from BALB/c mice as expected (Fig 1A and 1B).

However, real-time PCR measurement indicated that MRL and MRL/Lpr mice B cell BAFFR mRNA was expressed at similar levels as BALB/c cells (Fig 1C). Subsequent genetic analyses revealed a single nucleotide mutation, a cytosine to thymidine transition at position 130, in a conserved region of the N-terminus of BAFFR gene *TNFRSF13C*. This transition predicts a missense substitution of serine for proline (P44S) in the extracellular domain adjacent to the BAFF-binding site (Fig 1D and 1E) [36].

We then tested another commercially available anti-BAFFR antibody, clone 9B9, and found that, unlike clone 7H22-E16, it could bind MRL and MRL/Lpr B cells (Fig 1F). This result suggests that BAFFR is present on the surface of MRL and MRL/Lpr B cells, and that the epitope recognized by clone 7H22-E16 may be disrupted by the P44S mutation. While the anti-BAFFR 9B9 antibody was capable of binding the mutant BAFFR, significantly less antibody bound 2 month old MRL and MRL/Lpr B cells as compared to BALB/c (Fig 1F and 1G). Since anti-BAFFR 9B9 antibody is also functionally capable of blocking BAFF binding to BAFFR, and BAFFR mRNA expression is comparable between three strains, these results either indicate less surface expression of BAFFR on MRL strains, or steric hindrance by BAFFR bound BAFF in MRL/Lpr and MRL mice [37].

**Fig 2:**
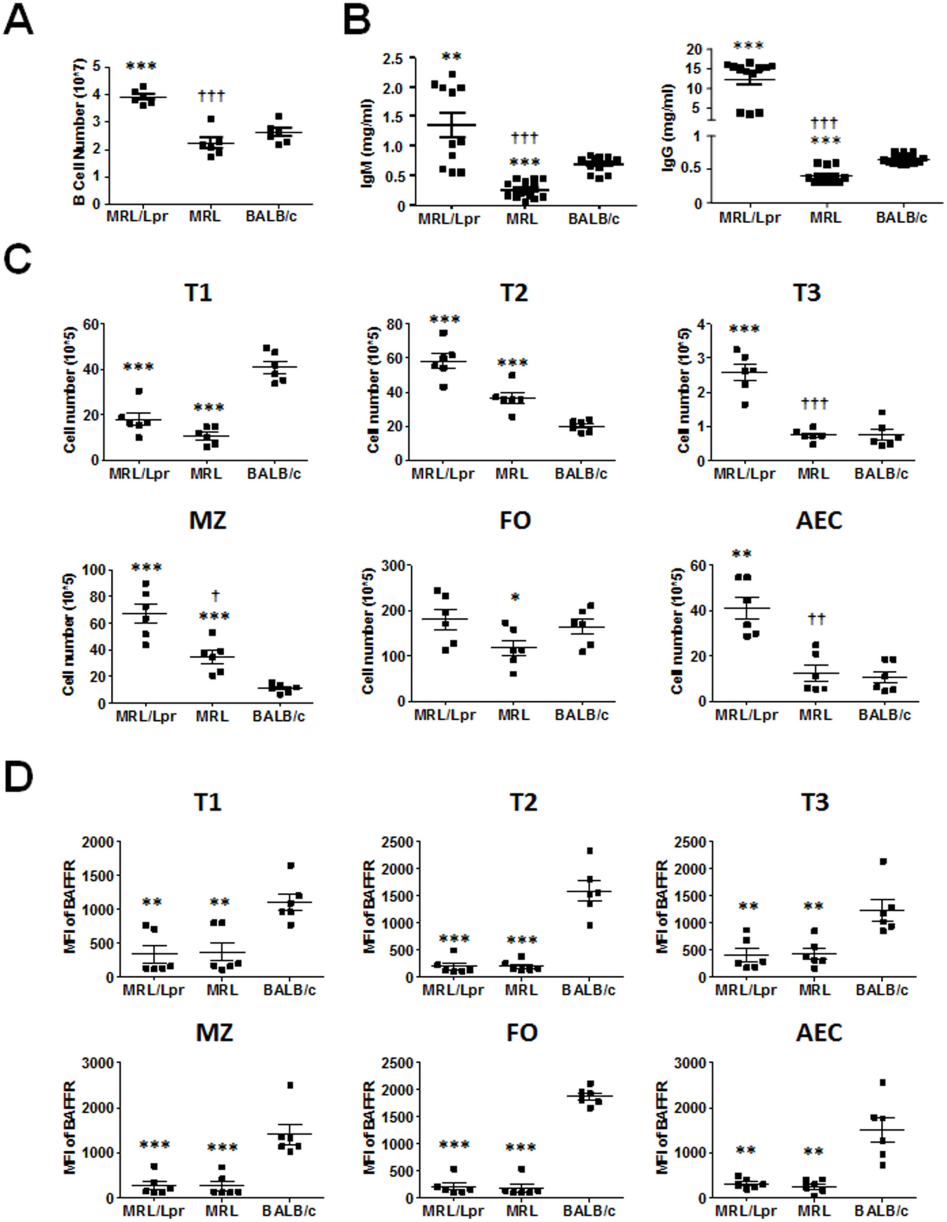
Absolute B cells numbers and the characterization of BAFFR expression on B cell subsets in 2 month old mice. (A) Graph of mean number ± SD of splenic B cells (CD19^+^) determined by flow cytometry (n = 6 mice per strain). (B) Concentrations of total IgM and IgG were determined by ELISA. Mean ± SD of sera IgM and IgG concentrations from 11 to15 female mice are shown. (C-D) B cells (CD19^+^) were gated for splenic B cell subsets based on CD93, IgM and IgD expression, using the previously described gating strategy published by Uslu and colleagues [34]. Transitional B cell populations are defined as T1 (CD93^+^IgM^+^IgD^-^), T2 (CD93^+^IgM^+^IgD^+^), and T3 (CD93^+^IgM^-^IgD^-^). Mature B cell populations are defined as MZ (CD93^-^IgM^+^IgD^-^), FO (CD93^-^IgM^+^IgD^+^), and AEC (CD93^-^IgM^-^IgD^-^). (C) Graphs of mean number ± SD of splenic B subset from 6 mice per strain as determined by flow cytometry are shown. (D) MFI ± SD of BAFFR on B cells subsets was determined by flow cytometry. Data shown are from 6 mice per group. * p < 0.05, ** p < 0.01, and *** p < 0.001 indicate statistically significant differences between MRL/Lpr or MRL vs BALB/c, while † p < 0.05, †† p < 0.01, and ††† p < 0.001 indicate statistical differences between MRL and MRL/Lpr strains.

The absence of BAFF-BAFFR signaling has been reported to cause the loss of peripheral B cells subsets such as transitional (T), follicular (FO), marginal zone (MZ), and antigen experienced cells (AEC) such as memory cells, plasma blasts, plasma cells, and isotype switched cells [38, 39]. Therefore, we sought to determine whether the P44S BAFFR mutation impacted spleen cellular composition and B cell numbers. Young, 2 month old, MRL/Lpr mice spleen weight and cell numbers were significantly more than MRL and BALB/c mice (S1 Fig). Similarly, MRL/Lpr mice spleen contained significantly more B cells than MRL and BALB/c mice spleens (Fig 2A). MRL mice splenic B cells were less than BALB/c mice but this difference did not reach statistical significance.

The reduced number of B cells in MRL mice is supported by significantly lower total sera IgM and IgG (Fig 2B). However, MRL/Lpr mice, which also carry the P44S mutation had elevated B cell counts as well as significantly higher total sera IgM and IgG than MRL and BALB/c mice. The differences between MRL/Lpr and MRL strains is likely due to the resistance of MRL/Lpr mice B cells to Fas-mediated apoptosis [2, 5].

Next, we sought to determine whether the low number of B cells and reduced sera antibodies in MRL mice was due to a defect in B cell maturation. Transitional B cell populations; T1 (CD93^+^IgM^+^IgD^-^), T2 (CD93^+^IgM^+^IgD^+^), and T3 (CD93^+^IgM^-^IgD^-^) as well as mature B cell populations; MZ (CD93^-^IgM^+^IgD^-^), FO (CD93^-^IgM^+^IgD^+^), and AEC (CD93^-^IgM^-^IgD^-^), were enumerated from the spleens of young, 2 month old mice (Fig 2C). MRL/Lpr and MRL mice had significantly reduced numbers of T1 cells compared to BALB/c mice. Conversely, both the MRL strains had significantly higher number of T2 cells than BALB/c mice. The number of cells in MRL/Lpr mice T3 subset was significantly more than MRL and BALB/c mice subsets. As expected from a lupus-prone mouse, the frequency of MZ B cells and AEC were higher in MRL/Lpr mice then the other two strains [40]. MRL mice MZ cells were significantly higher than those of BALB/c mice, while BALB/c mice FO cells were significantly higher than MRL mice FO cells. The fact that both MRL strains had lower number of T1 cells indicate either a defect in the maturation of newly immigrating B cells from the bone marrow or a defect in B cell differentiation from precursor cells in the bone marrows of P44S BAFFR expressing mice. However, the fact that MRL/Lpr mice had significantly higher numbers of T2, T3, MZ and AEC B cells than WT BAFFR expressing BALB/c B cells suggests that Fas signaling may be important for controlling autoreactive B cells at both transitional and more mature stages. BAFFR was expressed on all B cell subsets analyzed (Fig 2D). Interestingly, MRL/Lpr and MRL mice BAFFR expression was significantly lower than BALB/c mouse on all subsets tested.

### Survival is impaired in BAFFR P44S expressing B cells while signaling remains intact

**Fig 3:**
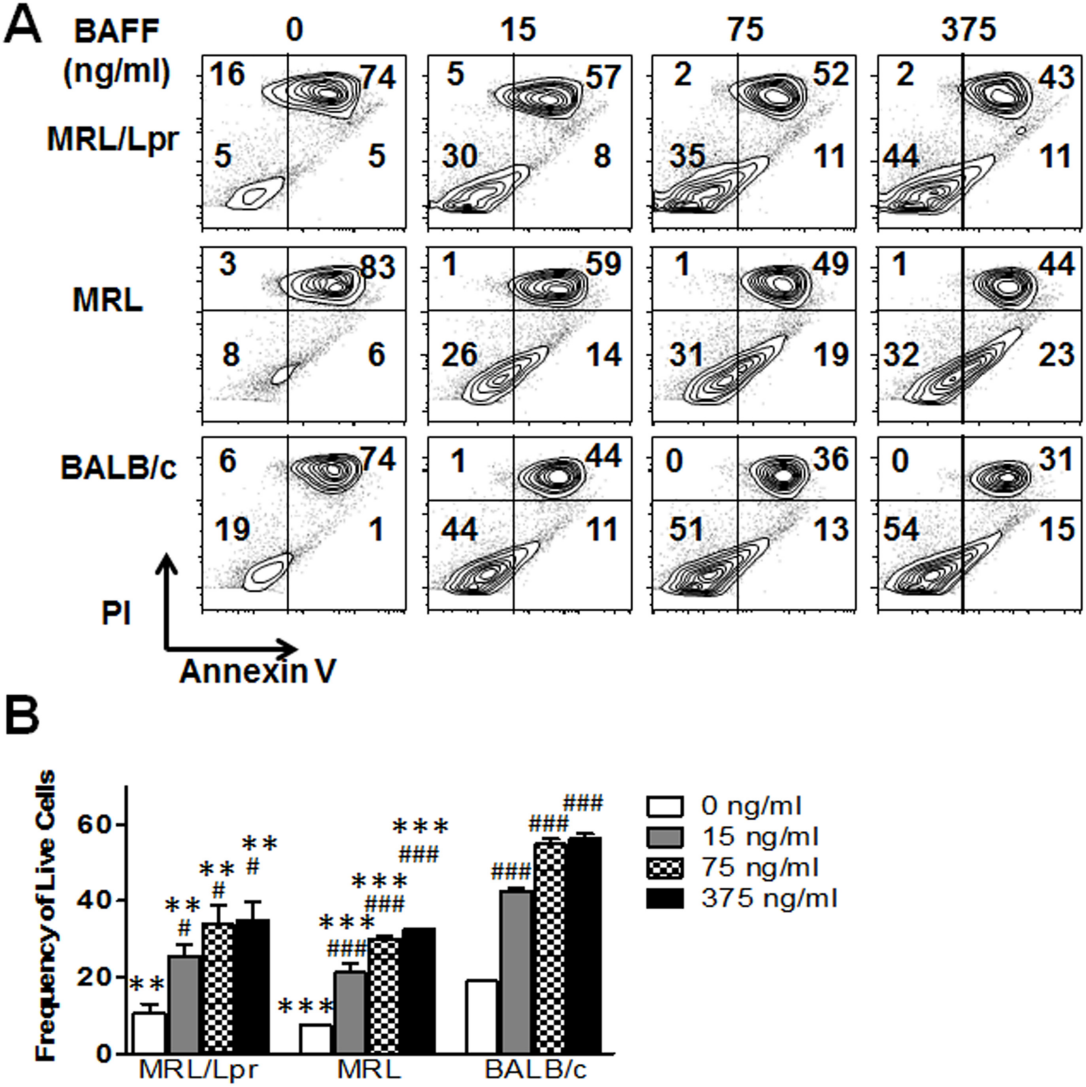
Evaluation of B cell survival in 2 month old mice. (A) Flow cytometry sorted B cells were incubated for 2 days in culture with increasing concentrations of BAFF (0, 15, 75, or 375 ng/ml). Cell apoptosis was measured in flow cytometry by staining with propidium iodide (PI) and annexin V. Annexin V^+^PI^-^ cells are in early apoptosis, annexin V^+^PI^+^ are in late apoptosis, and annexin V^-^PI^-^ are live cells. (A) Shown bi-exponential plots of PI vs annexin V are data from one representative experiment out of three. (B) Mean live cell frequency ± SD from three experiments are plotted. ** p < 0.01 and *** p < 0.001 indicate statistically significant differences between MRL/Lpr or MRL vs BALB/c, while # p < 0.05 and ### p < 0.001, indicates statistical differences within a strain induced by BAFF treatment.

We next sought to identify functional differences that may be caused by the P44S mutation. Since BAFFR has been shown to mediate BAFF-induced B cell survival [28], we evaluated the ability of BAFF to attenuate apoptosis in BAFFR P44S expressing B cells. As expected, after 48 hours of culture, a majority of B cells in media alone from all three strains were in late stages of apoptosis, defined by co-staining of annexin V and propidium iodide (annexin V^+^PI^+^) (Fig 3A).

Compared to BALB/c mouse, B cell survival (annexin V^-^PI^-^) was significantly reduced in the BAFFR P44S-expressing MRL and MRL/Lpr B cells. Though the MRL and MRL/Lpr B cells showed a higher rate of spontaneous apoptosis, stimulation with BAFF improved B cell survival in these strains, indicating that the mutant BAFFR P44S is functional.

**Fig 4:**
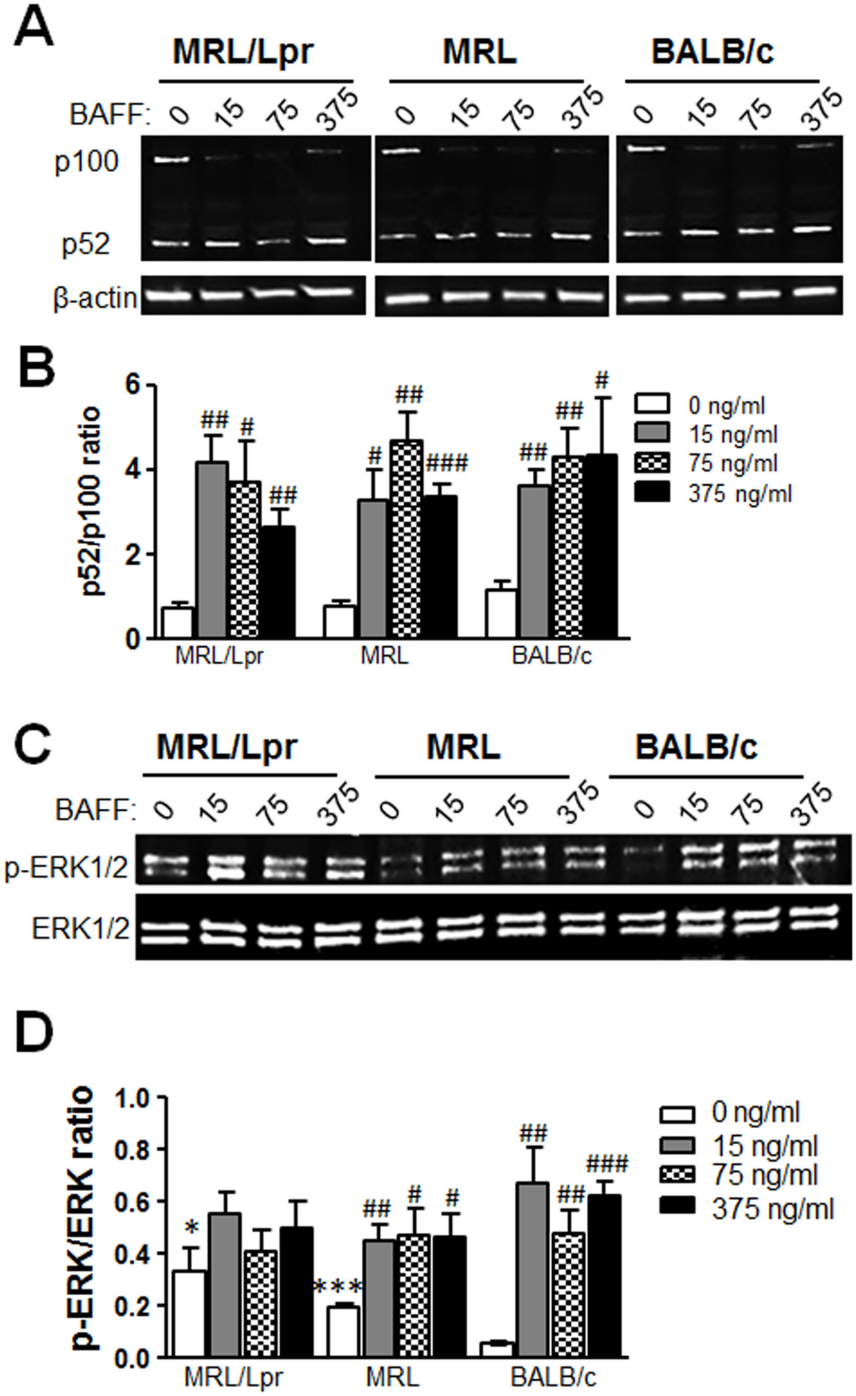
Activation of alternative NF-κB and ERK1/2 pathways in BAFF stimulated BAFFR P44S expressing B cells from 2 month old mice. Flow cytometry sorted B cells from MRL/Lpr, MRL, and BALB/c mice were incubated for 24 hours in culture with increasing concentrations of BAFF (0, 15, 75, or 375 ng/ml). (A) NF-κB2 (p100) processing to p52 was assessed by Western blot analysis of whole B cell extracts using β-actin as a loading control. One out of three experiments with similar results is shown. (B) Mean p52/p100 ratio ± SD from three independent experiments as determined by Western blot analysis is plotted. (C) Total and phosphorylated (p)-ERK1/2 molecules were detected in Western blot analysis. One out of four experiments with similar results is shown. (D) The ratio of the band density of p-ERK1/2 molecules to total ERK1/2 was calculated for each condition. Mean p-ERK/ERK ratio ± SD from four experiments are shown. * p < 0.05 and *** p < 0.001 indicate statistically significant differences between MRL/Lpr or MRL vs BALB/c, while # p < 0.05, ## p < 0.01, and ### p < 0.001 indicate statistical differences within a strain induced by BAFF treatment.

Functional studies demonstrated that P44S mutated BAFFR was still capable of mediating cell survival. Nevertheless, the fact that the frequency of live B cells in MRL strains was less than the BALB/c mouse after BAFF stimulation (Fig 3B) warranted further analysis of BAFFR mediated signaling. To determine BAFFR mediated signaling, B cells were stimulated with varying concentrations of BAFF (0, 15, 75, or 375 ng/ml) and NF-κB2 processing of the gene product p100 to generate p52 was assessed by Western blot [33]. B cells from all three strains significantly activated NF-κB2 processing upon BAFF stimulation at every concentration of BAFF used with no significant differences among strains (Fig 4A and 4B).

BAFF-induced activation of NF-κB2 pathway alone is not sufficient for B cell survival; a sustained ERK1/2 MAPK activation is also required [28, 41]. Schweighoffer and colleagues recently reported that CD79a and BCR function as adaptor proteins for BAFFR [41]. As such, B cells die rapidly in the absence of this tonic B cell signal mediated by BAFFR/BCR/CD79a complex. Persistent ERK1/2 activation by BAFFR suppresses the accumulation of pro-apoptotic Bcl-2 family member BIM, saving the cell from apoptosis [27, 28]. We determined ERK1/2 activation by measuring the level of ERK1/2 phosphorylation (p-ERK1/2) in B cells after 24 hours of culture with increasing concentrations of BAFF (Fig 4C). As expected, BALB/c B cells responded with a significant increase in p-ERK1/2 levels upon BAFF stimulation (Fig 4D). Phosphorylated ERK1/2 levels were significantly elevated in MRL and MRL/Lpr strains even before BAFF stimulation. This constitutive ERK1/2 phosphorylation did not significantly increase further in MRL/Lpr B cells after BAFF stimulation, whereas MRL B cells responded to BAFF.

**Fig 5:**
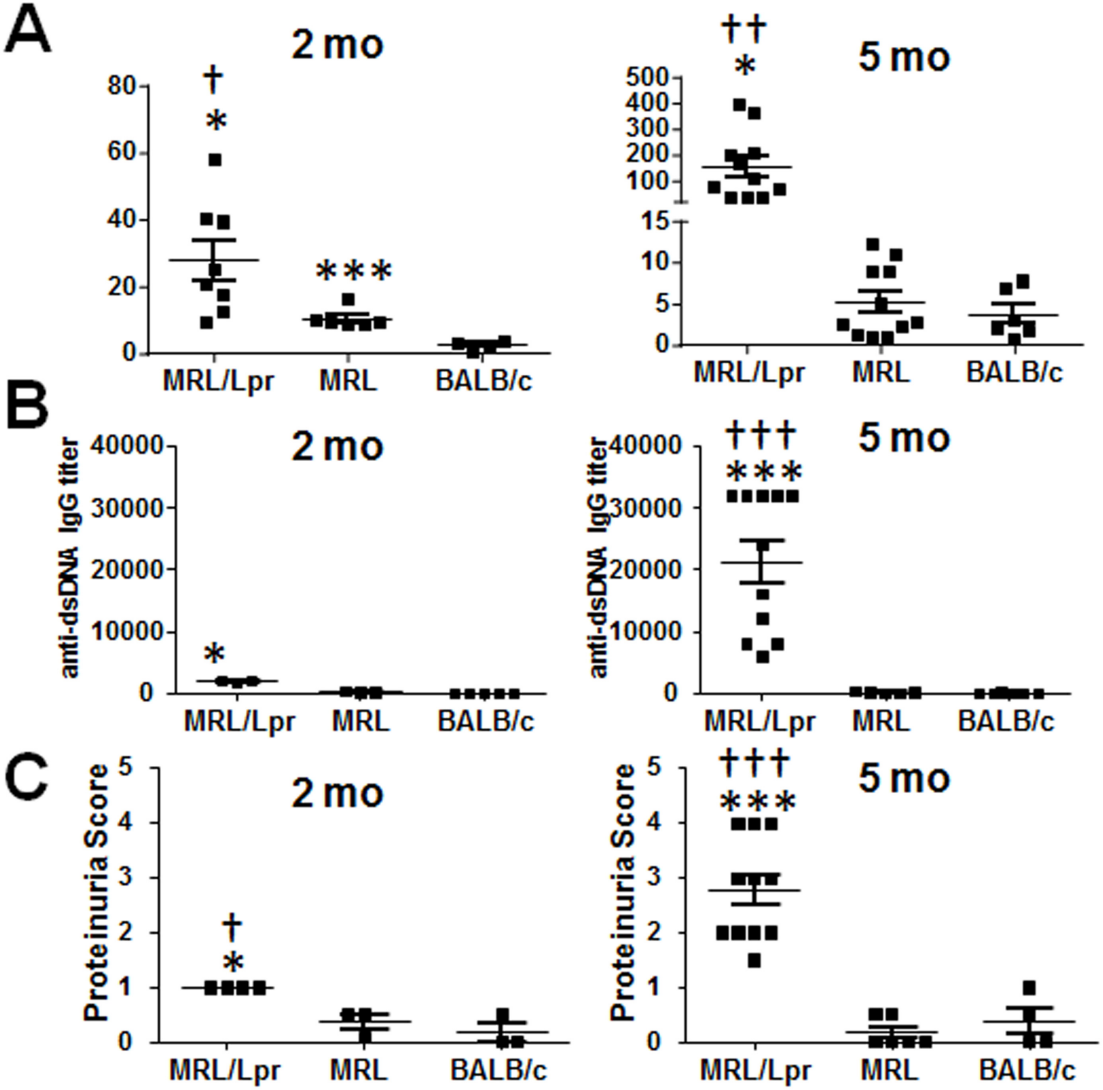
MRL/Lpr mice with SLE have elevated sera BAFF, elevated anti-dsDNA antibodies and kidney disease. (A) Mean ± SD of serum BAFF levels determined by ELISA. The data shown are from 5 to13 female mice per group. (B) Titers of anti-dsDNA IgG in serum are plotted. Sera from 2 and 5 month old mice were tested for anti-dsDNA IgG levels by ELISA. Titer is defined by the serum dilution giving an OD reading 2 times higher than background. Mean titers ± SEM from 3 to11 mice in each group were plotted. (C) Mean ± SD of proteinuria score is plotted. Approximate levels of protein levels in urine: score 0 = 0- trace, score 1 = 30 mg/dL, score 2 = 100 mg/dL, score 3 = 300 mg/dL, and score 4 = > 2000 mg/dL. The data shown are from 5 to13 female mice per group. * p < 0.05, ** p < 0.01, and *** p < 0.001 indicate statistically significant differences between MRL/Lpr or MRL vs BALB/c, while † p < 0.05, †† p < 0.01, and ††† p < 0.001 indicates statistical differences between MRL and MRL/Lpr strains.

Both P44S BAFFR strains had significantly higher levels of p-ERK1/2 in non-treated cells than BALB/c mice, which may be caused by previous in vivo exposure to BAFF or some other ERK1/2-inducing stimulant (Fig 4D). All experiments in this study so far were performed on young (2 month old) mice to minimize the contribution of chronic inflammation in MRL/Lpr mice when characterizing the P44S BAFFR mutation. However, in both mice and humans the onset of disease was shown to be accompanied by increased levels of circulating BAFF and APRIL [7, 11, 13, 14]. At the same time, overexpression of BAFF was sufficient to cause lupus-like disease in mice [10]. Consistent with these previous studies, although markedly less than the BAFF levels in 5 month old mice, both autoimmune susceptible MRL and MRL/Lpr strains had higher levels of serum BAFF than BALB/c mice by 2 months of age, (Fig 5A).

This early exposure to BAFF in vivo in MRL and MRL/Lpr strains may account for the differences in BAFFR signaling and induction in survival. Excessive BAFF signaling in vivo may have desensitized B cells to further stimulation in vitro. IgG autoantibodies specific to dsDNA were detected in two month old MRL/Lpr mice at significantly higher levels than control mice but levels were still low (Fig 5B). As expected, anti-dsDNA antibody levels increased further in 5 month old MRL/Lpr mice as the disease progressed. Another indicator of SLE is kidney disease triggered by IgG and C3 deposition within glomeruli, which can be assessed by proteinuria levels [5]. Urinalysis results showed that MRL/Lpr mice have significantly higher levels of proteinuria at both 2 and 5 months of age (Fig 5C).

### BAFFR expression in MRL/Lpr mouse after the onset of disease

**Fig 6:**
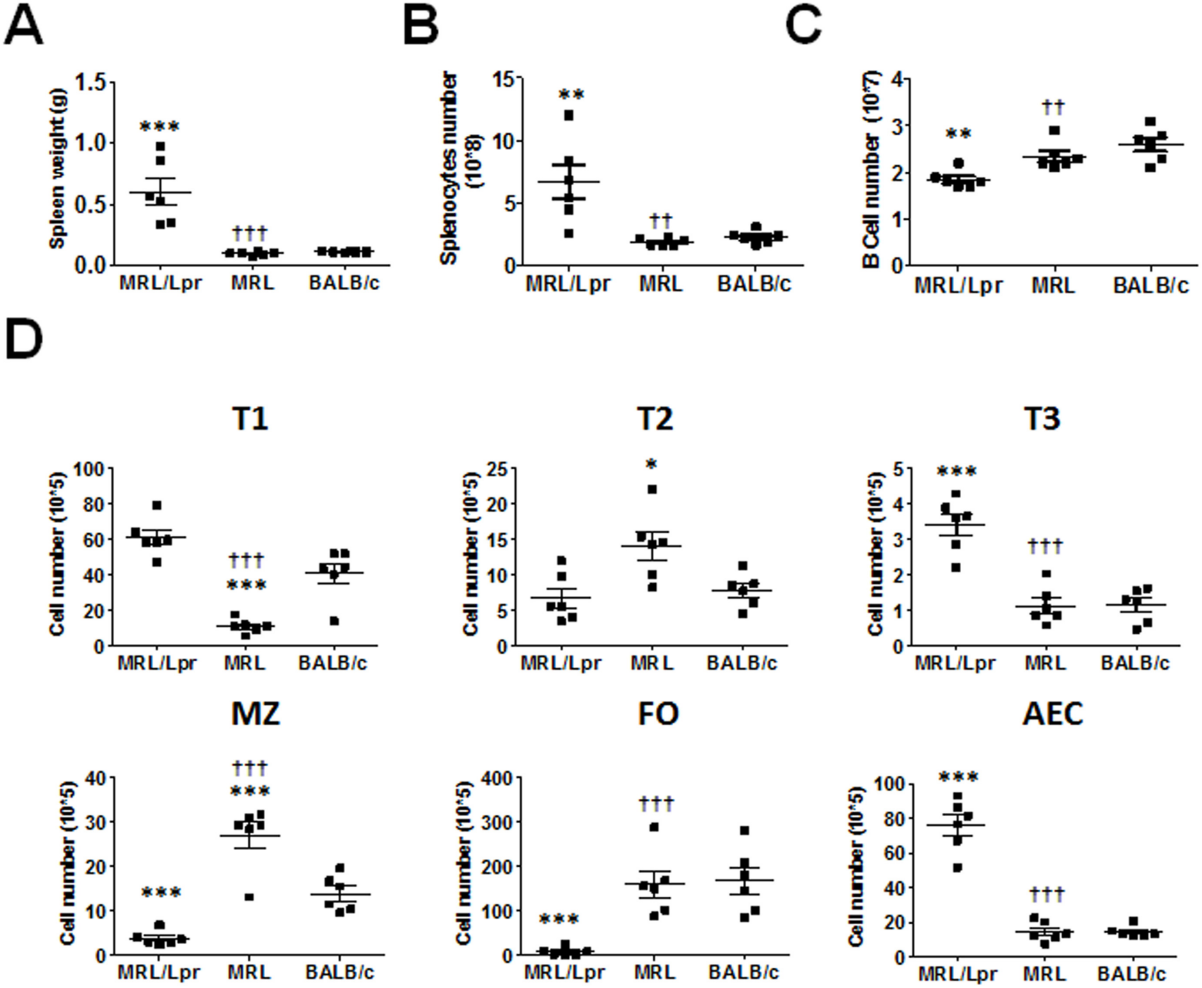
Absolute B cell and B cell subset numbers in 5 month old mice. (A) Mean ± SD spleen weight for each strain is plotted (n = six mice per strain). (B) Graph of mean number ± SD of total splenocytes and (C) splenic B cells (CD19^+^) determined by flow cytometry (n = 6 mice per strain). (D) Splenic B cells (CD19^+^) were gated for B cell subsets T1, T2, T3, MZ, FO and AEC as described in Fig 2. * p < 0.05, ** p < 0.01, and *** p < 0.001 indicate statistically significant differences between MRL/Lpr or MRL vs BALB/c, while †† p < 0.01, and ††† p < 0.001 indicate statistical differences between MRL and MRL/Lpr strains.

Next, we assessed the changes in splenic B cells and BAFFR expression in 5 month old MRL/Lpr mice with lupus disease. Splenic weight of MRL/Lpr mice was significantly increased (Fig 6A) compared to the splenic weight of 2 month old mice (S1 Fig).

The increase in MRL/Lpr mice splenic weight was accompanied by an increase in the number of splenocytes (Fig 6B). However, the number of B cells in MRL/Lpr mice was significantly less than the MRL and BALB/c mice B cell numbers (Fig 6C). The composition of B cell subsets was also different at 5 month (Fig 6D) as compared to 2 month old MRL/Lpr mice (Fig 2C). At 5 months, MRL/Lpr mice had higher numbers of T1 and T2 B cells than MRL mice (Fig 6D). Interestingly, the numbers of T2 cells were less in all three mouse strains at 5 months as compared to 2 month old mice. MRL/Lpr mice maintained a significantly larger T3 B cell population compared to both MRL and BALB/c mice. The most dramatic change in 5 month old mice B cell subsets were in the mature B cell subsets. The numbers of MZ and FO B cells were severely reduced at 5 months in MRL/Lpr mice as compared to MRL and BALB/c mice. The numbers of MZ and FO B cells in 5 month old MRL/Lpr mice were also significantly less than the 2 month old mice numbers. The majority of the B cells in MRL/Lpr were AEC.

**Fig 7:**
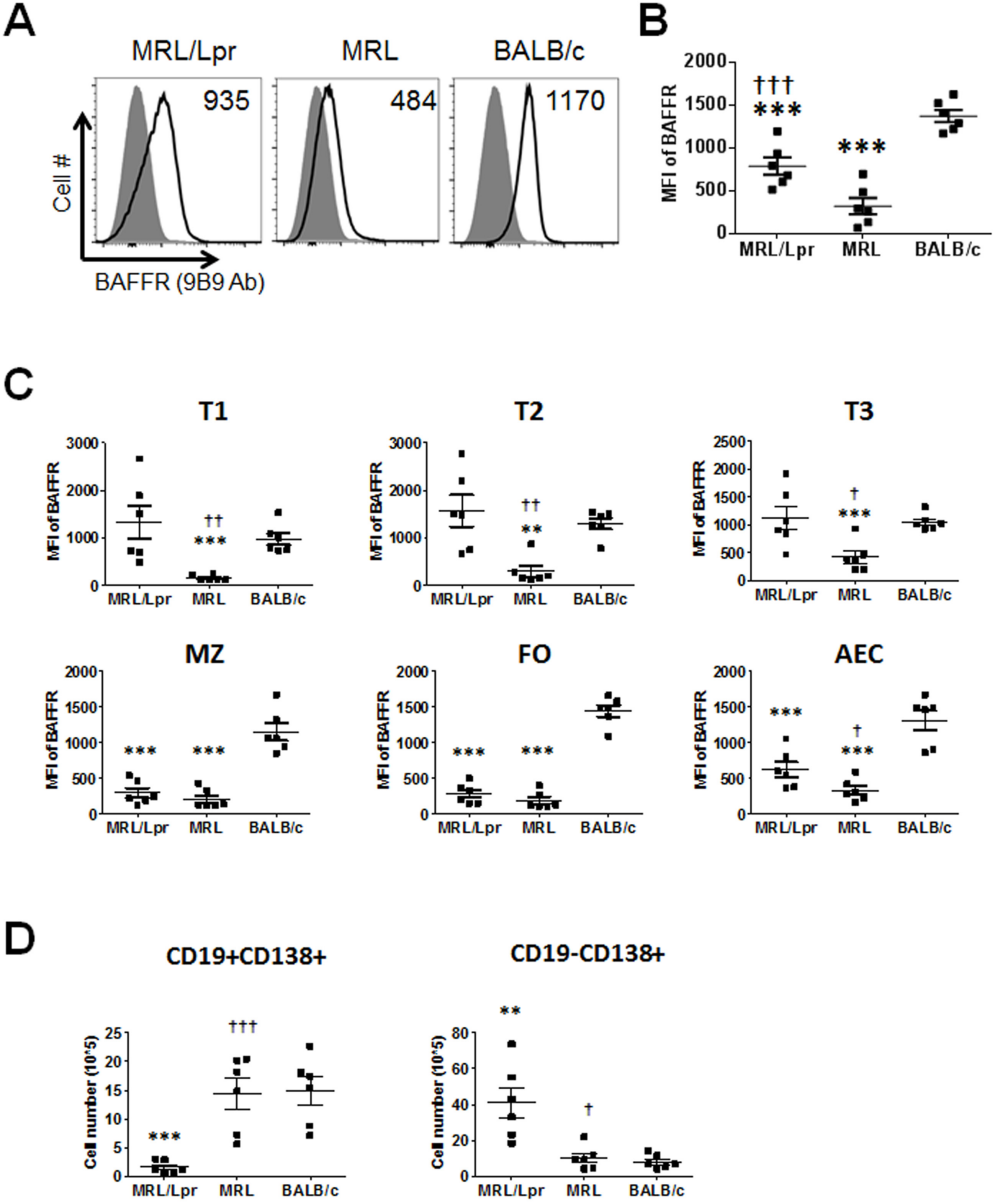
Characterization of B cell surface BAFFR in 5 month old MRL/Lpr mice. (A) Histograms of BAFFR expression on CD19^+^ splenic B cells determined by flow cytometry using the monoclonal antibody clone 9B9. Filled area shows isotype control antibody and open line indicates the intensity of staining for BAFFR. Representative data from each strain are shown. The mean fluorescence intensity (MFI) of B cells expressing BAFFR is indicated in each histogram. (B) MFI ± SD of BAFFR expressing B cells determined by flow cytometry is plotted. Data shown are from 6 female mice per group. (C) Splenic B cells (CD19^+^) were gated for B cell subsets T1, T2, T3, MZ, FO and AEC as described in Fig 2. MFI ± SD of BAFFR on B cells subsets was determined by flow cytometry. Data shown are from 6 mice per group. (D) Spleen ASC subsets, CD19^+^C138^+^ and CD19^-^CD138^+^, were analyzed by flow cytometry and graphs of mean number ± SD of splenic B cell subsets from 6 mice per strain were plotted.** p < 0.01, and *** p < 0.001 indicate statistically significant differences between MRL/Lpr or MRL vs BALB/c, while † p < 0.05, †† p < 0.01, and ††† p < 0.001 indicate statistical differences between MRL and MRL/Lpr strains.

As in 2 month old mice, both the 5 month old MRL strains expressed significantly lower levels of BAFFR on total B cells as compared to BALB/c mice B cells, except that MRL/Lpr mice had higher expression of BAFFR than MRL mice in 5 month old mice (Fig 7A and 7B).

The expression of BAFFR on 5 month old MRL mice B cell subsets was the same as the 2 month old MRL mice profile: BAFFR was expressed significantly less in MRL mice in comparison to BALB/c mice on all subsets (Fig 7C). In contrast and unlike in 2 month old mice, BAFFR expression on 5 month old MRL/Lpr mice T1, T2, and T3 subsets was not lower than those expressed on BALB/c mice subsets. Nevertheless, as in 2 month old mice, BAFFR was expressed less than BALB/c mice on MZ, FO and AEC subsets of MRL/Lpr mice. B cells typically lose surface BAFFR as they differentiate into antibody secreting cells (ASCs) [16, 23]. Indeed, staining of MRL/Lpr mouse splenic cells with CD19 and CD138 antibodies revealed that the fully differentiated plasma cells (CD19^-^CD138^+^) comprised the bulk of the AEC (Fig 7D).

### MRL/Lpr mice with established SLE have constitutive activation of NF-κB2

**Fig 8:**
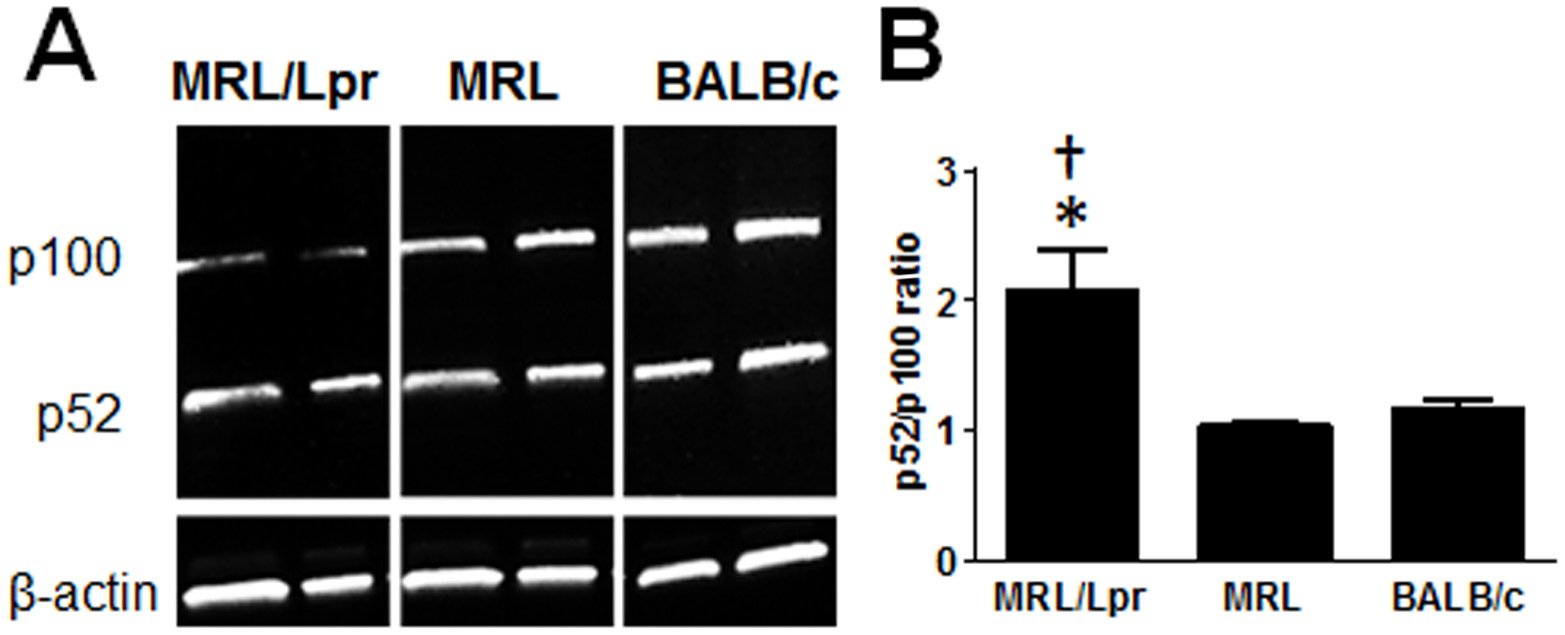
Evaluation of NF-κB2 pathway in 5 month old MRL/Lpr mice with active SLE disease. (A) Splenic B cells from MRL/Lpr mice with established SLE disease and age matched mice from MRL and BALB/c strains (5 to 6 mo) were analyzed for NF-κB2 signaling. After purification, B cells were rested for three hours in culture before assessing NF-κB2 processing from p100 to p52 in Western blot analysis. β-actin staining was used as a loading control. The data shown are from one representative experiment with 2 mice per strain. (B) Mean p52/p100 ratio ± SD from three independent experiments as determined by Western blot analysis. * p < 0.05 indicates statistically significant differences between MRL/Lpr vs BALB/c, while † p < 0.05 indicates statistical differences between MRL and MRL/Lpr strains.

In order to assess whether the further elevation of BAFF and increased ASC at 5 month lead to a higher degree of NF-κB2 activation in vivo, we measured p100 to p52 molecules in B cells. Western blot analysis revealed that aged MRL/Lpr mice B cells have significantly increased constitutive processing of NF-κB2 p100 to p52 compared to MRL and BALB/c B cells, likely the outcome of increased circulating BAFF (Fig 8A and 8B).

## Discussion

MRL mice develop late onset of SLE characterized by anti-dsDNA antibodies, the presence of immune complexes in the kidney, and pathophysiological changes associated with the deposition of immune complexes [9]. In MRL/Lpr mice, Fas deficiency induced by a mutation in the *Lpr* gene leads to a defect in apoptosis. Increased B cell survival is responsible for the lymphoproliferative disorder that induces a more severe form of SLE with early onset, resulting in about 50% mortality by 5 months of age [8, 9]. At the same time, mutated *Lpr* expression by C57BL/6 and C3H/HeJ mice does not lead to the development of SLE despite an increase in serum autoantibodies [42]. These studies are significant because they suggest that multiple genetic loci expressed by MRL mice may be conferring autoimmune susceptibility [2, 42–44]. Since BAFFR is critical for the selection and survival of B cells, it is a prominent candidate for promoting autoimmune susceptibility in B cells [20–22]. In this study, we report a novel mutation in the *TNFRSF13C* gene of MRL strains, which encodes for BAFFR. The BAFFR P44S mutation could have several possible immunopathological consequences. One possibility is constitutive signaling as seen in other autoimmune manifestations resulting from gain-of-function mutations [45, 46]. A constitutively activated BAFFR may rescue more autoreactive immature B cells from negative selection to become mature B cells capable of producing pathogenic autoantibodies [20]. A loss of function as a result of inefficient binding of BAFF to BAFFR would result in lower numbers of mature B cells as seen in BAFFR deficient mice [21]. A loss of function, but not a complete knock-out, may reduce the size of the B cell repertoire to the point where there is an excess BAFF per B cell allowing for more autoreactive B cells to mature [30, 47].

As shown in Fig 2, cell numbers in MRL mice B cell subsets were different than BALB/c mice for T1, T2, MZ and FO subsets. Similarly, MRL/Lpr mice T1, T2, T3, MZ and AEC subsets were significantly different than BALB/c mice subsets. In order to determine whether the difference between MRL strains and BALB/c mice B cell subset numbers is due to altered BAFFR signaling as a result of P44S mutation we evaluated the ability of BAFFR to respond to BAFF in several different assays. Assessment of B cell survival after BAFF stimulation indicated that MRL mice B cells respond to BAFF as efficiently as B cells from BALB/c mice since both these strains had comparable increases in the frequency of live cells. Compared to MRL mice, MRL/Lpr mice had reduced late transitional B cells counts but had elevated mature B cell counts and significantly higher total sera IgM and IgG than MRL and BALB/c mice. The differences among the P44S BAFFR stains are likely due to the Fas mutation in MRL/Lpr mice that reduces B cell apoptosis [2, 5]. Signaling experiments confirmed the intact BAFFR function in MRL and MRL/Lpr mice. BAFF stimulation induced NF-κB2 signaling in 2 month old MRL and MRL/Lpr B cells as efficiently as in B cells from BALB/c mice. Persistent ERK1/2 activation by BAFFR maintains protection from apoptosis [27, 48] by suppressing the accumulation of pro-apoptotic Bcl-2 family member BIM saving the cell from apoptosis [27, 28]. BAFF induced ERK1/2 signaling in MRL B cells to similar levels seen in BALB/c mice. Interestingly, both P44S BAFFR strains had significantly higher levels of constitutively activated ERK1/2 than BALB/c mouse. Although constitutive signaling may suggest gain-of-function, since BAFFR P44S mutation occurs in the extracellular domain near the BAFF-binding site, selective ERK1/2 MAPK constitutive induction due to P44S mutation without NF-κB2 p100 pathway involvement is unlikely. Instead, cross-reactive BCR engagement with self-antigen or other genetic susceptibility loci in this strain is likely responsible for B cell hyperactivity [49]. Collectively, these data indicate that the P44S BAFFR mutant is functional with no signaling defect.

Follicular B cells are the dominant form of naïve B cells in the spleen and we found that BAFFR is significantly lower on both young and older MRL and MRL/Lpr mice FO B cells than on BALB/c strain cells. It is possible that the reduced-surface expression measured on these cells may lead to an increased sensitivity to both BCR-mediated and spontaneous apoptosis. This is of interest, because it has been reported that autoreactive B cells have higher basal activation of their BCR yet lower surface BAFFR and consequently are more dependent on BAFF for survival [20]. These data are supported by multiple studies, which show an inverse correlation between BAFFR expression and disease severity accompanied by circulating BAFF levels [14, 50]. In this study, we also detected higher levels of serum BAFF in young MRL and MRL/Lpr mice, which continued to rise in MRL/Lpr mice over time. B cell surface BAFFR expression was significantly lower in older MRL/Lpr mice as they developed severe disease symptoms. The decrease in BAFFR level was mostly in mature B cell subsets MZ, FO and AEC. Low BAFFR expression is also reported in patients with SLE [14, 50]. A likely consequence of low BAFFR expression is higher engagement of TACI with excess BAFF, which in turn results in increased antibody secretion since TACI promotes plasma cell generation and immunoglobulin secretion [51]. Supporting this hypothesis, we found significantly elevated numbers of ASC in aged MRL and MRL/Lpr strains. Since BAFFR is predominately expressed by naïve and memory B cells and is down-regulated in ASCs [16, 23] it is likely that low BAFFR in aged MRL/Lpr mice is a result of increased BCR engagement and ASC differentiation. A functional consequence of this diminished BAFFR expression in ASCs is the inability of the anti-BAFFR antibody used in SLE therapy to impact the survival of bone marrow or splenic plasma cell [52]. In clinical trials, anti-BAFFR antibody therapy results in a decrease in plasmablasts along with naïve and activated B cell subsets, while preserving memory B cells [53]. In these patients the decrease in plasmablasts numbers is likely a result of a decrease in naïve and activated B cell pool, which supply plasmablast population with new cells. At the same time, the preservation of serum anti-pneumococcal and anti-TT antibodies after anti-BAFFR treatment is a reflection of the maintenance of memory and plasma cells that do not express BAFFR and therefore do not rely on BAFFR mediated survival signals.

In conclusion, there are known and still yet unidentified autoimmune susceptibility loci that contribute to the development of SLE in mice with the MRL background. Here, we identified a mutant form of BAFFR in MRL mice. However, no defect was observed in known signaling pathways associated with BAFFR activation. Finally, our flow cytometry experience indicates that when studying BAFFR expression in MRL strains, P44S mutation should be considered in the selection of anti-BAFFR antibodies.

## Supporting Information

S1 Fig Spleen weight and splenocyte counts in 2 month old mice. Mean ± SD spleen weight and total splenocytes for each strain is plotted (n = 6 mice per strain). *p < 0.05, and ** p < 0.01 indicate statistically significant differences between MRL/Lpr or MRL vs BALB/c, while, †† p < 0.01 indicates statistical difference between MRL and MRL/Lpr strains.
(PPT)
