# Supplementary figures and images for: MRL Strains Have a BAFFR Mutation without Functional Consequence
Source: PLoS One. 2016 May 5;11(5):e0154518. doi: 10.1371/journal.pone.0154518 (PMC4858247; doi:10.1371/journal.pone.0154518)

## Slide 1
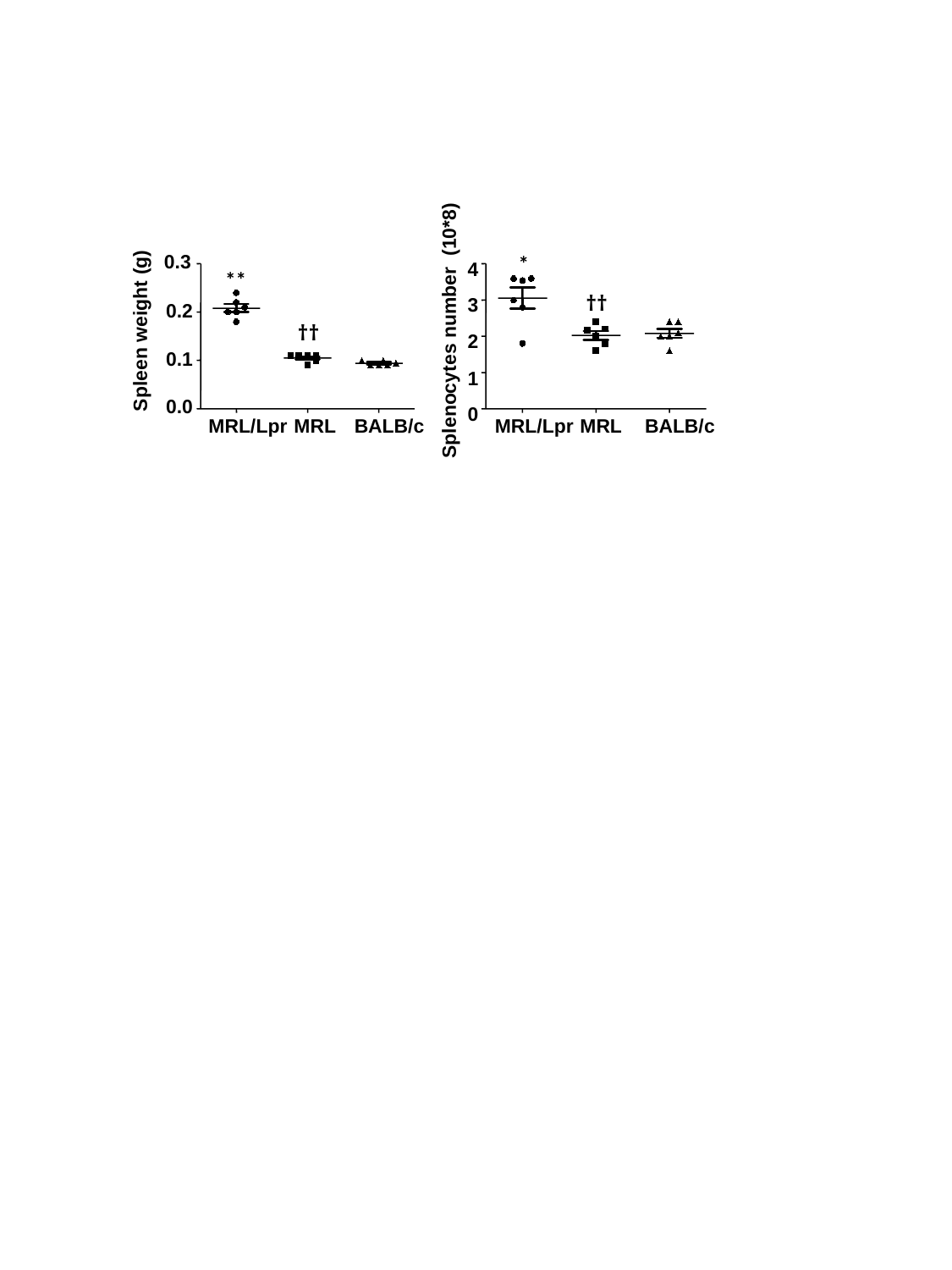

4
3
Splenocytes number (10*8)
2
1
0
MRL/Lpr
MRL
BALB/c
0.3
0.2
Spleen weight (g)
0.1
0.0
MRL/Lpr
MRL
BALB/c
*
**
††
††

Supplement: S1 Fig — Mean ± SD spleen weight and total splenocytes for each strain is plotted (n = 6 mice per strain). *p < 0.05, and ** p < 0.01 indicate statistically significant differences between MRL/Lpr or MRL vs BALB/c, while, †† p < 0.01 indicates statistical difference between MRL and MRL/Lpr strains. (PPT) [file pone.0154518.s001.ppt]
